# Optimization of Methods Verifying Volunteers’ Ability to Provide Hospice Care

**DOI:** 10.1007/s13187-016-1154-7

**Published:** 2016-12-14

**Authors:** Marta Szeliga, Jadwiga Mirecka

**Affiliations:** 1 Department of Medical Education, Faculty of Medicine, Jagiellonian University Medical College, ul. św. Łazarza 16, 31-530 Krakow, Poland; 2 St. Lazarus Hospice, Krakow, Poland

**Keywords:** Volunteer, Hospice, Method of verification

## Abstract

The subject of the presented work was an attempt at optimization of the methods used for verification of the candidates for medical voluntary workers in a hospice and decreasing the danger of a negative influence of an incompetent volunteer on a person in a terminal stage of a disease and his or her relatives. The study was carried out in St. Lazarus Hospice in Krakow, Poland, and included 154 adult participants in four consecutive editions of “A course for volunteers – a guardian of the sick” organized by the hospice. In order to improve the recruitment of these workers, the hitherto methods of selection (an interview with the coordinator of volunteering and no less than 50% of attendance in classes of a preparatory course for volunteers”) were expanded by additional instruments—the tests whose usefulness was examined in practice. Knowledge of candidates was tested with the use of a written examination which consisted of four open questions and an MCQ test comprising 31 questions. Practical abilities were checked by the Objective Structured Clinical Examination (OSCE). A reference point for the results of these tests was a hidden standardized long-term observation carried out during the subsequent work of the volunteers in the stationary ward in the hospice using the Amsterdam Attitude and Communication Scale (AACS). Among the tests used, the greatest value (confirmed by a quantitative and qualitative analysis) in predicting how a given person would cope with practical tasks and in contact with the sick and their relatives had a practical test of the OSCE type.

## Introduction

Involving volunteers in the care for patients and relatives of patients in the final stage of terminal illness constitutes a common standard in palliative care in Poland as well as in many other countries of Europe and the world. Hospice volunteers are people who perform various jobs and who are of a different background; what they have in common is their will to provide unpaid aid for those who need it [3].

Volunteering brings, above all, great benefits both for patients and volunteers themselves [1, 6]. There are, however, some articles whose authors warn that volunteering may manifest differently and bring about some dangers as well, which should not be ignored [8, 9]. Also, the author’s own experience over her thirteen-year work in a hospice has shown that some volunteers actually should not be ones. “Good will” is often nota sufficient basis for actions if real improvement of quality of life is expected in the recipients of these actions. Not everyone who applies to become a volunteer, especially a hospice volunteer, can become one. It is important to get to know not only candidates’ motivation but also to check their knowledge, skills, and attitudes. A vast majority of candidates for hospice volunteers do have good intentions, and the most commonly declared motivation is the will to provide aid. However, there are some factors that can disqualify candidates, such as a too weak psyche, lack of emotional balance, intellectual disability, or improper ethical attitudes. These elements are real threat and a potential source of harm done to the patient and to oneself.

In order to reduce the risk of incompetent volunteers’ actions on patients or their families, most Polish hospices provide special preparatory courses. However, a standardized system of preparing medical volunteers for hospice care has not been worked out. Presently, a lot of facilities (unfortunately, not all of them) have worked out clear principles of recruitment of volunteers. These principles look similar: a candidate who wants to undertake some actions involving a direct contact with the patient usually completes a preparatory course and is interviewed by the volunteer coordinator. Those who reveal behavioral disorders or unethical attitudes during the interview should not be approved. However, this criterion is subjective as it depends merely on the coordinator’s experience and sensitivity. In turn, individual palliative or hospice care facilities work out their own training for candidates for hospice volunteers, and such training programs prepared in individual facilities vary considerably from one to another. They are usually courses lasting a few to a few dozen hours of classes. They do not usually end with examinations. So far, a standardized selection method has not been developed. Such *status quo* is a common standard in Poland.

The fact that introduction of courses for candidates for volunteers and trainings for volunteer coordinators has become more common recently proves that the issue of selection while recruiting hospice volunteers has become an increasing problem [16].

The introduction of additional tests for candidates for medical volunteers in a hospice has not been intended to create formal barriers preventing individuals from undertaking this role. Its purpose was to protect the good of the patient and his family being under palliative care and also to protect the candidates for the volunteers themselves. Candidates who do not meet specific criteria may be suggested taking up some other forms of volunteer work. Thus, they may become volunteers of the administrative, maintenance, or fundraising staff, but not medical volunteers.

The methods of assessment of the candidates which were the subjects of this study were not new. They have been extensively used, described, and tested in many countries as effective methods employed at the recruitment of medical workers [4, 12] and include checking of their knowledge, practical abilities, interpersonal communication skills, attitudes, and some personality features. The novelty was their application for the candidates for voluntary medical workers of a hospice.

The study was carried out in the years 2010–2012 in St. Lazarus Hospice in Krakow, Poland.

## Aim of the Study

The subject of the presented work was the attempt at optimization of the methods used for verification of the candidates for medical voluntary workers in a hospice through the application of appropriate tools.

Research questions:

1. Are there any methods for prediction of functioning volunteers in practice?
2. Is there any possibility of application of methods for the assessment of volunteers, which is used for the evaluation of health care professionals?
3. Which from the investigated methods of evaluation is the most appropriate for volunteers’ verification?

## Methodology

For the study, a positive opinion of the Bioethics Committee of the Jagiellonian University had been obtained.

In order to improve the recruitment of volunteers to work with patients in the hospice, the hitherto methods of selection (“an interview with the coordinator of volunteering and no less than 50% of attendance in classes of a preparatory course for volunteers”) were supplemented by additional instruments—tests whose usefulness was examined in practice.

The knowledge of candidates was tested with the use of a written examination in the form of open questions (essay-type questions) and a multiple choice test (MCQ). Practical abilities were checked by the Objective Structured Clinical Examination (OSCE). A reference point for the results of these tests was a hidden standardized long-term observation carried out during the subsequent work of the volunteers in the stationary ward in the hospice.

The written exam comprising four essay-type questions was aimed at revealing attitudes of the future hospice volunteers. In order to obtain the possibly most objective opinion about a candidate, for each question, four evaluation criteria had been prepared. For each of the criteria from 1 to 5, points could be obtained (1—a response in terms of the criterion very bad or no, 5—a very good, extensive answer). For every question, volunteer could get a total of 4–20 points and for the whole test 16–80 points.

The MCQ consisted of 31 items; for each item, one correct answer should be chosen from among four possible. It was a test of knowledge acquired during the course. The questions were focused on the knowledge of principles of interpersonal communication, propagation of the idea of hospice care, dilemmas associated with patient care, and patients’ everyday problems.

The practical exam consisted of three 5-min OSCE stations with standardized patients (SPs), who displayed the same behavior and expressed the same needs, and each time responding in the same way towards each test taker. Next, on the basis of the strictly defined criteria, the SPs assessed the future volunteers’ abilities in regard to getting in touch, proper communication, attitudes towards the patient, respecting his or her dignity as well as the ability to recognize the patient’s needs, and providing aid relevant to these needs.

Stations 1 and 2 checked simple practical skills: assisting a weakened patient in walking and helping to change the underwear involving a bedridden patient. The last station checked the ability to communicate, with a psychologist acting as an SP.

Before the examination, all the test takers received exactly the same initial information about the objectives and general principles of the exam and a briefing sheet comprising a detailed description of the organization of the examination with the requirements for test takers.

The research tool used during the long-term observation was the Amsterdam Attitude and Communication Scale (AACS) [5] modified for the purpose of this study. Accordingly, the assessment focused on the following areas:

1. patient and his family (ability to communicate),
2. hospice workers and other volunteers (ability to cooperate),
3. volunteering (dutifulness, practical skills),
4. own person (ability to do a critical self-evaluation; the will to acknowledge and correct one’s mistakes).

For greater validity of the prepared examinations, both the questions and the tasks for the volunteers as well as the evaluation criteria had been consulted with a clinical psychologist and with an experienced volunteer coordinator. In 2009, we conducted a pilot study covering 27 volunteers. As a result of these studies, the MCQ was extended by six additional questions, two questions were changed, and a more detailed checklist for the OSCE station no. 3 was created.

The study involved 154 people who had completed the course and decided to take the final tests. From among all the participants, 141 people (91.5%) were made subject to a hidden standardized long-term observation in the inpatient ward of the hospice. Within this group, 136 people went through all the phases of the study. The remaining five participated only either in the theoretical (*N* = 3) or practical (*N* = 2) exam.

## Statistical Analysis of Data

For the statistical analysis we used *SPSS 14 PL software.*

The results of the essay-type questions were expressed in a number of obtained points. The MCQ was presented as a percentage of the correct answers. In the case of the OSCE, a subjective impression of the observer was presented next to objective assessment expressed in a number of obtained points. The relationship between objective and subjective evaluation in the context of this test was examined with the use of Spearman’s rank correlation (r_sp_).

To find out which of the tests can be the best indicator of the usefulness of the candidates, the results of these tests were compared with a hidden standardized long-term observation, which was a confirmation of the readiness of a volunteer to cope with practical tasks. The dependence between the results of particular tests and the result of an observation was measured with the r_sp_ because of the lack of normality of distribution of the variables studied.

Due to the fact that a surprisingly high turnover of volunteers was observed during the study, the authors also decided to find out whether the test results could help to predict how long a given person will contribute to hospice volunteering (Mann-Whitney *U* test for two independent samples was used). The duration was divided into two categories: volunteer work being longer or shorter than 4 months.

## Results

The results of all the used forms of examinations correlated significantly positively (bilateral statistical significance was *p* < 0.0001) with the results of a hidden standardized long-term observation (Table 1). The strongest relation was shown by the result of practical tests of the OSCE type. Both the objective evaluation obtained on the basis of the criteria from the check list (r_sp_ = 0.67) and subjective impressions of the observer-simulated patient (r_sp_ = 0.62) correlated higher with the observation result than the result of the written examination with open questions (*p* = 0.48) or the results of the MCQ (r_sp_ = 0.44) cf. Table 2.

**Table 1:** Correlation between the result of a hidden standardized long-term observation and the results of other tests

Type of test	Hidden standardized long-term observation	
	Correlation	Statistical significance (*p*)
MCQ	r_sp_ = 0.44	*p* < 0.0001
Essay-type questions	r_sp_ = 0.48	*p* < 0.0001
OSCE	r_sp_ = 0.67	*p* < 0.0001
The overall result of all three tests	r_sp_ = 0.69	*p* < 0.0001

*r*_*sp*_ Spearman’s rank correlation

**Table 2:** Correlation between objective evaluation and subjective assessment of each of the used stations of the OSCE and the results of a hidden standardized long-term observation

The practical exam OSCE type		Hidden standardized long-term observation	
		Correlation	Statistical significance (*p*)
Objective evaluation	Station 1	r_sp_ = 0.46	*p* < 0.0001
	Station 2	r_sp_ = 0.53	*p* < 0.0001
	Station 3	r_sp_ = 0.49	*p* < 0.0001
	OSCE (overall rating)	r_sp_ = 0.67	*p* < 0.0001
Subjective assessment	Station 1	r_sp_ = 0.38	*p* = 0.9606
	Station 2	r_sp_ = 0.50	*p* < 0.0001
	Station 3	r_sp_ = 0.39	*p* = 0.8406
	OSCE (overall rating)	r_sp_ = 0.62	*p* < 0.0001

*r*_*sp*_ Spearman’s rank correlation

The total results of all three forms of tests indicated higher correlation with the result of prolonged observation (r_sp_ = 0.69) than any particular tests separately. However, this correlation was on a level comparable with the one obtained by the practical exam OSCE alone.

Assessment of each of the used stations of the OSCE type separately also correlated higher with practical coping of the volunteer with patient’s care than a multiple choice test. The written examination with open questions obtained slightly higher coefficient of correlation (r_sp_ = 0.48) with standardized observation as compared to the first station (r_sp_ = 0.46), but lower than the remaining stations.

Particular forms of examinations correlated positively with each other, and each of these correlations was statistically significant Table 3. The MCQ showed stronger relation with open questions in a written examination (r_sp._ = 0.46; *p* < 0.0001) and weaker with a practical test of the OSCE type (r_sp_ = 0.24; *p* = 0.0040). Open questions showed positive correlation on a level similar to all employed forms of assessment (with the OSCE r_sp_ = 0.44; *p* < 0.0001/with a MCQ r_sp._ = 0.46; *p* < 0.0001).

**Table 3:** Correlations between particular forms of examinations

	MCQ	Essay-type questions	OSCE
MCQ		r_sp._ = 0.46, *p* < 0.0001, *N* = 151	r_sp_ = 0.24, *p* = 0.0040, *N* = 145
Essay-type questions	r_sp._ = 0.46, *p* < 0.0001, *N* = 151		r_sp_ = 0.44, *p* < 0.0001, *N* = 145
OSCE	r_sp_ = 0.24, *p* = 0.0040, *N* = 145	r_sp_ = 0.44, *p* < 0.0001, *N* = 145	

*r*_*sp*_ Spearman’s rank correlation, *p* statistical significance, *N* number of subjects

According to the performed qualitative analysis, the practical test of the OSCE type turned out to have significantly higher prognostic value than both theoretical tests. Moreover, in two cases, it revealed the candidates’ features which precluded their undertaking of the work with the sick-fresh, not suppressed emotions after the loss of a close person in one of the female candidates and an aggressive attitude towards simulated patients in one of the male candidates. Carrying out this type of an exam allowed, therefore, to avoid unnecessary trauma by both potential candidates for volunteers and by the persons whom they would take care of while working in the stationary ward in the hospice

The scores obtained in the theoretical tests, both in the MCQ and the open questions tests, did not reflect in any way the duration of the subsequent involvement in hospice volunteer work. People who did better in the OSCE and obtained higher marks were involved in volunteer work for a long time significantly more often (cf. Tables 4 and 5). As for this test, only the objective score was a significant predictor of the duration of volunteer work; this correlation was not observed for the SP’s subjective impression.

**Table 4:** The relationship between the results of the applied testing methods and the duration of the subsequent involvement in hospice volunteer work (volunteer work being longer or shorter than 4 months)

Type of test	Volunteer work being *shorter* than 4 months			Volunteer work being *longer* than 4 months			*p*
	*N*	Median	X + SD	*N*	Median	X + SD	
MCQ^a^	103	77.4	74.42 ± 9.89	49	80.0	76.33 ± 9.45	0.2649
Essay-type questions	102	70	66.93 ± 10.29	49	72	69.00 ± 9.01	0.2042
OSCE	98	86	83.74 ± 14.18	49	92	89.08 ± 14.01	0.0161
long-term observation	91	77	75.71 ± 10.51	50	80	81.46 ± 8.32	0.0013

*N* number of subjects, *X* arithmetic average, *SD* standard deviation, *p* statistical significance

^a^The result is given in percentage of correct answers

**Table 5:** Correlations between the duration of the subsequent involvement in hospice volunteer work and the results of other tests

Type of test	Volunteer work being *longer* than 4 months		
	*N*	r_sp_	*p*
MCQ^a^	152	0.09	0.2663
Essay-type questions	151	0.10	0.2052
OSCE	147	0.20	0.0155
OSCE—subjective impression of the SP’s	147	>0.01	0.9947
long-term observation	141	0.27	0.0011

*N* number of subjects, *r*_*sp*_ Spearman’s rank correlation, *p* statistical significance sided

^a^The result is given in percentage of correct answers

People who received higher scores during the long-term observation, coping better with volunteer work in the ward, were also involved in it substantially longer.

## Discussion

The presented study is the first attempt in Poland to find a standardized tool that could be useful for verification of hospice volunteers.

While searching for suitable tools, the authors focused on the factors minimizing the risk of negative impact of the hospice on the volunteer and vice versa: impact of a volunteer on the recipients of their care, such as proper knowledge, skills, and ethical attitudes [7, 10, 11] the last being the most difficult to verify in the initial selection phase.

In the world literature, no similar studies have been found, whose subjects were hospice volunteers. However, the results of a similar study involving medical students were published, which were to check whether the exams taken may reliably predict medical staff’s subsequent clinical performance [2, 14, 15].

The obtained results can be compared to those published by Wilkinson et al. in 2004 [15]. They examined, among others, the correlation between the general assessments of doctors’ clinical performance in a hospital ward and the score they had obtained in the final exam of their medical studies. The results of this study confirmed—as in the presented study—that OSCE is the best predictor (from among the three forms of testing: 3-h written test consisting of open questions requiring short or long answers, MCQ consisting of 220 items, and a practical OSCE comprising eighteen 5-min stations).

Also, the results obtained in the presented study show that supplementing the practical exam with the theoretical part in two different forms of a written test increases its prognostic value in a very limited way. To raise the validity of this exam, it seems to be more reasonable to increase the number of OSCE stations than to introduce different forms of testing. With each subsequent station, the correlation coefficient increased. On the other hand, the scores of the individual stations separately correlated higher with the results of the long-term observation than the MCQ score. In the case of Stations 2 and 3, the correlations were also higher than for the open questions written test, which obtained a slightly higher correlation coefficient with the results of the standardized observation only in comparison to Station 1. Thus, taking into consideration the recommendation not to burden candidates too heavily, it seems reasonable to introduce even one of the OSCE stations as a complementary tool accompanying the methods of verification used until now.

In the abovementioned Wilkinson’s study, the correlation of the total score of the OSCE and the written MCQ (open questions were answered by another group of students) with the results of the clinical performance observation (0.64) was also at a level similar to the level of the correlation of OSCE only (0.63) with the observation.

The results of individual exams obtained in this study correlated significantly positively not only with the result of the long-term observation but also with each other. Thanks to this, on the basis of the mark obtained as a result of using one of the methods of testing, the result of a subsequent test taken by a given person can be predicted with great probability.

In the study published in 2005, Auewarakul et al. [2] obtained slightly different correlations between the exams used. The most significant difference was the fact that MCQ showed the highest correlation with OSCE in contrast to the present study, where the correlation of MCQ with OSCE was the lowest. In turn, a similarity may be the fact that the standardized mark obtained during the practice in a ward, like in this study, correlated higher with OSCE than with MCQ.

Based on the similarities between the results of the forms of testing the candidates for hospice volunteers used in this study and the results of similar exams used with medical students, a conclusion can be drawn that the empirically tested methods used for selecting medical staff may be used successfully, and after having been modified, they can be applied for selection of volunteers.

On the basis of the above shown analysis of usefulness of the particular forms of testing, the subsequent conclusion can be drawn: OSCE seems to be the most valuable tool for selecting volunteers from among the instruments used in the study. Its correlation with the subsequent clinical performance, despite using a shortened form of examination (three stations), was at a similar or even higher level than that obtained in the studies in which its extended version was used. Naturally, the authors of the study would not be tempted to claim that this exam is the best possible tool for verification of volunteers, or that it could be used as a completely independent tool. For this reason, they suggest that the practical OSCE could be a valuable complement of methods used so far and not the only selection tool.

Should carrying out OSCE in the form used in the study prove to be too challenging (the most difficult exam to carry out), it will be better, as the correlations between individual test results show, to use any other verification method than to resign completely from the assessment ending the preparatory training for future volunteers. In addition, the studies by Ramreje et al. [13] prove that medical students who were evaluated at the end of a lecture (irrespective of the method) remembered its contents remarkably better and for a longer time than those who did not undergo evaluation, irrespective of the students’ individual learning strategies.

It seems to be more reasonable, however, to introduce an examination resembling just one OSCE station than carrying out theoretical tests. Assuming that interpersonal skills and psychological preparation play the key role in preparing a volunteer to work with patients while selecting a single OSCE station, examiners should choose one that checks, above all, the ability to communicate with the patient and/or his family and not, e.g., manual skills. At such a station wrong stereotypical thinking, bias, religious fanaticism, lack of empathy are revealed most frequently, which were confirmed by the qualitative analysis of the OSCE type exam.

### Study Limitations

It must be remembered that volunteers’ future performance cannot be predicted with absolute certainty and clarity.

Setting up the optimal cutoff value for the volunteers might be a big problem if something that clearly discriminates them, which was observed in the case of two individuals during the study, namely, aggression manifested in relation to the simulated patient in the case of one man and a strong experience of fresh mourning after the loss of a husband in the case of one woman, does not take place.

### Clinical Implications

The procedure of verification of volunteers expanded with a standardized tool such as OSCE, apart from benefits for patients and volunteers themselves, may have a positive influence on the organization of work in a ward, which could be attributed to the fact that volunteers of higher competences would work with the terminally ill and because of a lower turnover of volunteers. OSCE was the best predictor of both volunteers’ clinical performance and the duration of their subsequent work as a hospice volunteer.

Setting up the optimal cutoff value requires further research, so that exam results could be recognized in the categories of “pass” or “fail”.

## Conclusions

1. The empirically tested methods used for selecting medical staff may be used successfully for selection of hospice volunteers.
2. A practical test of the OSCE type has a greater value for prediction of functioning volunteers in practice than open questions and MCQ.
3. Even introducing an exam, resembling the single OSCE station could be a valuable instrument significantly increasing the value of the procedure of verification of volunteers for work in a hospice.
